# Traditional landmark versus ultrasound guided tracheal puncture during percutaneous dilatational tracheostomy in adult intensive care patients: a randomised controlled trial

**DOI:** 10.1186/s13054-014-0514-0

**Published:** 2014-09-18

**Authors:** Máté Rudas, Ian Seppelt, Robert Herkes, Robert Hislop, Dorrilyn Rajbhandari, Leonie Weisbrodt

**Affiliations:** Royal Prince Alfred Hospital, Intensive Care Services Missenden Road, Camperdown, NSW 2050 Sydney, Australia; Nepean Hospital, Intensive Care Unit Derby St, Penrith, NSW 2750 Sydney, Australia; Sydney Nursing School - The University of Sydney 88 Mallett St, Camperdown, NSW 2050 Sydney, Australia; The George Institute for Global Health Level 13, 321 Kent St, NSW 2000 Sydney, Australia; Australian School of Advanced Medicine 2 Technology Place, Macquarie University, NSW 2109 Sydney, Australia

## Abstract

**Introduction:**

Long-term ventilated intensive care patients frequently require tracheostomy. Although overall risks are low, serious immediate and late complications still arise. Real-time ultrasound guidance has been proposed to decrease complications and improve the accuracy of the tracheal puncture. We aimed to compare the procedural safety and efficacy of real-time ultrasound guidance with the traditional landmark approach during percutaneous dilatational tracheostomy (PDT).

**Methods:**

A total of 50 patients undergoing PDT for clinical indications were randomly assigned, after obtaining informed consent, to have the tracheal puncture procedure carried out using either traditional anatomical landmarks or real-time ultrasound guidance. Puncture position was recorded via bronchoscopy. Blinded assessors determined in a standardised fashion the deviation of the puncture off midline and whether appropriate longitudinal position between the first and fourth tracheal rings was achieved. Procedural safety and efficacy data, including complications and number of puncture attempts required, were collected.

**Results:**

In total, 47 data sets were evaluable. Real-time ultrasound guidance resulted in significantly more accurate tracheal puncture. Mean deviation from midline was 15 ± 3° versus 35 ± 5° (*P* = 0.001). The proportion of appropriate punctures, defined *a priori* as 0 ± 30° from midline, was significantly higher: 20 (87%) of 23 versus 12 (50%) of 24 (RR = 1.74; 95% CI = 1.13 to 2.67; *P* = 0.006). First-pass success rate was 20 (87%) of 23 in the ultrasound group and 14 (58%) of 24 in the landmark group (RR = 1.49; 95% CI = 1.03 to 2.17; *P* = 0.028). The observed decrease in procedural complications was not statistically significant: 5 (22%) of 23 in the ultrasound group versus 9 (37%) of 24 in the landmark group (RR = 0.58; 95% CI = 0.23 to 1.47; *P* = 0.24).

**Conclusions:**

Ultrasound guidance significantly improved the rate of first-pass puncture and puncture accuracy. Fewer procedural complications were observed; however, this did not reach statistical significance. These results support wider general use of real-time ultrasound guidance as an additional tool to improve PDT.

**Trial registration:**

Australian New Zealand Clinical Trials Registry ID: ACTRN12611000237987 (registered 4 March 2011)

**Electronic supplementary material:**

The online version of this article (doi:10.1186/s13054-014-0514-0) contains supplementary material, which is available to authorized users.

## Introduction

Patients in intensive care frequently receive tracheostomy for long-term ventilator support. Percutaneous dilatational tracheostomy (PDT) has established advantages and is the preferred method over surgical tracheostomy [1], which is now reserved for select cases. Although overall complication rates are low, PDT remains one of the few procedures routinely undertaken in intensive care where significant adverse events, including death, are still reported [2-5]. With the increasing availability of bedside ultrasonography in the intensive care setting, preprocedural and real-time intraprocedural ultrasound guidance have been advocated as potential tools to further improve the safety and efficacy of the procedure [6,7].

Despite an increasing number of publications in the recent literature describing favourable results and advocating the use of ultrasound for PDT [8,9], there is a paucity of high-quality evidence. A recent systematic review failed to identify any randomised controlled trials in support of this modality [10]. To answer the question whether using real-time ultrasound guidance improves the procedural safety and efficacy of tracheal puncture, we conducted a prospective randomised controlled trial comparing this novel approach to the landmark method in adult intensive care patients requiring PDT.

## Methods

### Trial design

The TARGET Study (Traditional landmArk versus ultRasound Guided Evaluation Trial) was a prospective randomised controlled trial carried out at two participating sites: Royal Prince Alfred Hospital and Nepean Hospital in Sydney, Australia. The aim of the study was to compare the use of real-time ultrasound guidance to landmark-guided tracheal puncture during PDT. The primary outcome measure was the accuracy of tracheal puncture, defined as less than 30° deviation from midline and appropriate longitudinal puncture between the first and fourth tracheal rings. A secondary measure of efficacy was the first-pass success rate. Studied measures of safety were periprocedural and intermediate-term complication rates.

### Sample size

Lacking published data, we estimated the degree of malposition to calculate sample size requirements. Presuming an average displacement of 25° from midline in the landmark group and 15° in the ultrasound group with 15° and 10° respective standard deviations, we estimated that a sample size of 50 would be required to detect a statistically significant difference with a 0.05 confidence interval and statistical power over 80%.

### Participants and enrolment

Ethical approval was obtained for both participating sites (see Acknowledgements), and the trial is registered with the Australia and New Zealand Clinical Trials Registry (ACTRN12611000237987). Eligible adult intensive care patients were defined as being older than 18 years of age at the time of enrolment and requiring a percutaneous dilatational tracheostomy for clinical reasons, with the only exclusion criterion being pregnancy. Patients were unable to provide consent; therefore, in accordance with the ethical approval, informed consent was obtained from the person responsible prior to study enrolment. As set out by New South Wales health policy, the person responsible was most commonly an enduring guardian, a spouse or another close relative. Patients were randomised in a 1:1 ratio to the intervention and control arms using permuted blocks of four and six, and allocation concealment was maintained by using sequentially numbered, opaque, sealed envelopes created according to published recommendations [11]. An independent member of the research team who was not directly involved with the study in any other capacity created the randomisation sequence using a computer algorithm [12] and prepared and sealed the envelopes. Once consent was obtained, patients were enrolled by selecting the topmost sequentially numbered envelope, which contained the study group allocation.

### Interventions

Patients in both groups underwent a standard PDT using the Ciaglia single tapered dilator technique [13] using the Ciaglia Blue Rhino kit (Cook Medical, Bloomington, IN, USA). Percutaneous tracheal needle puncture was followed by insertion of a guidewire, use of the tapered dilator to create the tract and insertion of the tracheostomy tube. The study intervention was use of real-time ultrasound guidance to guide the tracheal needle puncture. A Sonosite M-Turbo portable ultrasound machine (FUJIFILM SonoSite, Bothell, WA, USA) or a Siemens Acuson Cypress portable ultrasound machine (Siemens, Munich, Germany) was used with 5-MHz linear transducers and sterile ultrasound probe covers (Bard Access Systems, Salt Lake City, UT, USA). A midline longitudinal probe position was used to identify the cricoid and tracheal rings in cross-section, thereby allowing us to determine the required level of puncture, ideally between the first and fourth tracheal rings. This was followed by scanning the neck in a transverse probe position and identifying the thyroid and cricoid cartilages, tracheal rings, thyroid gland and isthmus, and the carotid and jugular vessels bilaterally. If significant midline vascular structures were noted, the planned puncture position was modified accordingly. Tracheal puncture was carried out using a previously described transverse probe position and real-time out-of-plane technique [7]. An additional movie file depicts the procedure in more detail (see Additional file 1). Printed pamphlets with normal ultrasound anatomy in the longitudinal and transverse planes and a description of the study procedure were available for reference. In the control arm, palpation of anatomical landmarks was used to carry out the tracheal puncture, which is normal practice at the participating institutions. Mandatory bronchoscopy using Pentax EPK-1000 (Pentax Medical, Montvale NJ, USA) or Olympus (Olympus America, Center Valley, PA, USA) video bronchoscopes was carried out after guidewire insertion to confirm intraluminal position before dilation and to document the puncture position for analysis. Bronchoscopy had to take place only after the guidewire was inserted. Bronchoscopy during needle puncture is not considered standard practice in either of the participating units and was not permitted in the study protocol. The analogue video output from the bronchoscope tower was captured with a BlackMagic analogue-to-digital video converter (BlackMagic Design, Fremont, CA, USA) to record the full duration of the bronchoscopy from insertion of the bronchoscope into the endotracheal tube until its removal. Video was recorded using an Apple MacBook Pro laptop computer (Apple, Cupertino, CA, USA) and BlackMagic video recording software (BlackMagic Design). Dilation of the tract and insertion of the tracheostomy tube were carried out in both groups as per normal practice. All procedures were carried out by intensive care specialists or supervised senior trainees in intensive care medicine, which is consistent with current Australian practice. All proceduralists were familiar with the landmark approach, but had varying experience with the ultrasound-guided technique. To capture a representative group of clinicians, no proceduralists were excluded, as long as they were willing to learn and perform both the study and control procedures.

### Data collection and outcome measures

We collected baseline demographic data and severity of illness indicators (Acute Physiology and Chronic Health Evaluation II on admission and Sequential Organ Failure Assessment scores on the day prior to tracheostomy) (Table 1). Data collected during and immediately after the procedure included number of passes, with subsequent passes defined by the need to withdraw the needle completely from the skin and reinsert it, and immediate periprocedural complications, defined as complications arising during the procedure or during the following 1 hour (Table 2). A postinsertion anteroposterior mobile chest radiograph was mandatory, and any complications such as pneumothorax were noted. Video acquired during bronchoscopy was recorded as described above. Follow-up of the patients was carried out until day 90 or decannulation, whichever occurred first. Time to wean from the ventilator, ICU length of stay and time to decannulation were recorded. Any tracheostomy-related adverse events during the follow-up period were documented (Table 2).

**Table 1:** **Baseline characteristics** ^**a**^

**Characteristics**	**Landmark (** ***N*** **= 25)**	**Ultrasound (** ***N*** **= 25)**	***P*** **-value**
Age (yr), mean (SD)	58.4 (15.2)	57.0 (15.1)	0.748
Males, *n/N* (%)	12/25 (48%)	12/25 (48%)	1.000
Weight (kg), mean (SD)	87.8 (25.5)	75.2 (19.3)	0.059
BMI (kg/m^2^), mean (SD)	30.3 (8.4)	26.1 (7.2)	0.080
APACHE II score, mean (SD)	22.7 (5.6)	22.3 (6.6)	0.807
Days ventilated prior to PDT, mean (SD)	10.1 (4.5)	9.3 (4.7)	0.546
SOFA score on day prior to tracheostomy, mean (SD)	4.3 (2.2)	3.9 (2.6)	0.516
PaO_2_/FiO_2_ ratio ≤200, *n*/*N* (%)	4/24 (16)	9/23 (39)	0.085
INR, mean (SD)	1.1 (0.1)	1.1 (0.2)	0.849
APTT (seconds), mean (SD)	39.8 (9.9)	35.3 (9.0)	0.107
Indication for tracheostomy, *n/N* (%)			
Respiratory failure	18/25 (72)	16/25 (64)	0.544
Poor neurological status	7/25 (28)	9/25 (36)	0.544

^a^The groups were similar at baseline. APACHE II, Acute Physiology and Chronic Health Evaluation II; APTT, Activated partial thromboplastin time; BMI, Body Mass Index; INR, International Normalised Ratio; PaO_2_/FiO_2_, Partial pressure of arterial oxygen to fraction of inspired oxygen; PDT, Percutaneous dilatational tracheostomy.

**Table 2:** **Follow-up data and procedural and intermediate-term complications** ^**a**^

**Complications**	**Study groups**		***P*** **-value**
**Procedural complications,** ***n*** **(%)** ^**b**^	**Landmark (** ***N*** **= 24)**	**Ultrasound (** ***N*** **= 23)** ^**c**^	
Patients with procedural complications^b^	9 (37%)	5 (22%)	0.237
Patients with complications excluding minor bleeding^b^	2 (8%)	2 (9%)	0.965
Only minor bleeding/no intervention	7 (29%)	3(13%)	0.177
Bleeding requiring intervention	2 (8%)	0 (0)	0.157
Pneumothorax	0 (0)	0 (0)	1.000
Tracheal injury	0 (0)	0 (0)	1.000
Oesophageal injury	0 (0)	0 (0)	1.000
Paratracheal placement	1 (4%)	0 (0)	0.322
Haemodynamic instability	1 (4%)	0 (0)	0.322
Desaturation	1 (4%)	1 (4%)	0.975
Ruptured ETT cuff	0 (0)	1 (4%)	0.302
**Intermediate-term complications,** ***n*** **(%)**	**Landmark** **(** ***N*** **= 24)**	**Ultrasound (** ***N*** **= 24)** ^**c**^	
Accidental decannulation	0 (0)	1 (4%)	0.322
Pressure ulcer	0 (0)	1 (4%)	0.322
Bleeding	0 (0)	0 (0)	1.000
Soft-tissue infection	0 (0)	0 (0)	1.000
Follow-up			
Days to wean	9.0	8.0	0.448
Days to decannulation	19	24	0.819
ICU length of stay	23	22	0.556

^a^ETT, Endotracheal tube. ^b^Patients with multiple procedural complications were counted as one in the overall procedural complication rate. ^c^In the ultrasound group, procedural data were missing for one patient and follow-up data were available for all 24 patients.

### Data analysis

**Figure 1:**
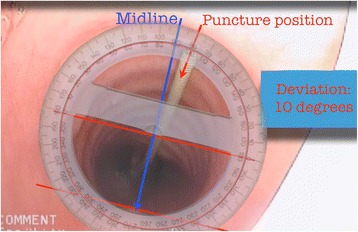
**Measuring puncture deviation from the tracheal midline.** Aligning the protractor with the anterior tracheal wall at the level of the puncture, followed by rotating so that the transverse axis is parallel with the posterior tracheal wall, defines the geometrical anterior tracheal midline. Deviation of the puncture was then determined in degrees.

The primary outcome measures of accurate craniocaudal positioning and midline deviation of the tracheal puncture were derived from the bronchoscopic recordings by two blinded assessors with extensive clinical experience in bronchoscopy and percutaneous tracheostomy insertion. Still images showing the entire tracheal lumen and the guidewire entering the trachea were taken from the video recordings. Images were scaled up or down to standardise the tracheal lumen size at the level of the guidewire entry (10 cm in transverse diameter) to allow uniform assessment. Aspect ratios were maintained, and there was no additional postprocessing of images. The two blinded assessors used an Apple iPad (Apple) with preloaded images and a standard translucent 360° protractor (Cellco, China). The geometric anterior tracheal midline was defined by aligning the protractor with the anterior curve of the trachea at the level of the puncture and aligning the transverse axis with the posterior tracheal wall. Using the aligned protractor, we recorded the deviation from the midline in degrees (Figure 1). This step was followed by determination of the craniocaudal position, based on the bronchoscopic images. This was deemed either appropriate, too caudal or too cranial, depending on whether the puncture site was between the first and fourth tracheal rings or above or below these, respectively. The complete bronchoscopic video recordings were also available for the assessors to review on the tablet device. If there was a greater than 10° disagreement between the assessors or there was disagreement in terms of craniocaudal positioning, they were asked to review the images in question together to form a consensus. All statistical analyses were carried out using Prism for Mac OS X v6.0 (GraphPad Software, La Jolla, CA, USA). Deviation from the midline was assessed using an unpaired *t*-test. Deviation was also analysed with dichotomised data, *a priori* defining midline ±30° as appropriate and deviation beyond this level as inappropriate. Evaluation of the above data, along with assessment of longitudinal placement and number of multiple-pass instances between the groups, was evaluated using a χ^2^ test.

## Results

**Figure 2:**
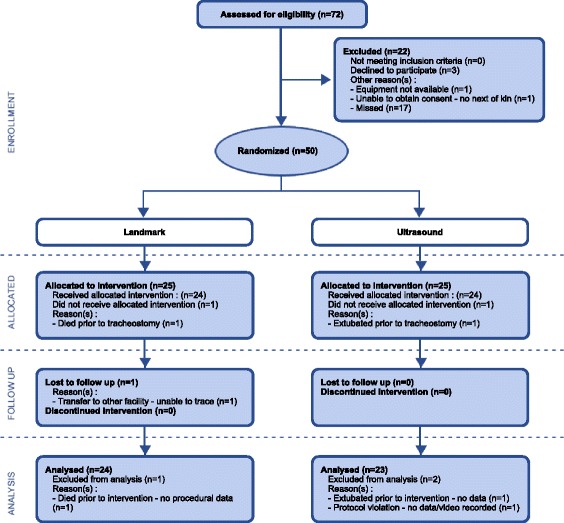
**CONSORT flowchart.** CONSORT, Consolidated Standards of Reporting Trials.

During the course of the 11-month recruitment period between August 2011 and July 2012, there were 72 eligible patients, from among whom 55 were screened and 50 were enrolled, with 25 patients in each group (Figure 2). No patient met the exclusion criteria, and no patient was deemed to require a surgical tracheostomy. No patient was found to have significant aberrant pretracheal vasculature in the ultrasound group. The groups were similar at baseline (Table 1). One patient had undergone recent cervical spine surgery, and two patients had previous tracheostomy scars. Twenty-four patients in each group received the allocated intervention. One patient in the landmark group died after randomisation and before the tracheostomy was performed, and one person in the ultrasound group made an unexpected recovery after randomisation and could be extubated without the need for tracheostomy. Procedural data were missing for one patient in the ultrasound group because of a protocol violation, resulting in no recording being made. Follow-up data were complete for all but one patient in the landmark group, who was lost to follow-up after transfer to another institution and therefore the final decannulation date is unknown. All data were analysed according to intention to treat.

In both groups, one-third of the procedures were carried out by intensive care specialists and the remainder by senior trainees under supervision. Mean midline deviation in the ultrasound and landmark groups was 15 ± 3° versus 35 ± 5° (*P* = 0.001), with a difference of 20 ± 6° (95% CI = 8.0 to 31.8). Appropriate midline puncture, defined *a priori* as the anterior one-third of the tracheal ring, or midline ±30°, was achieved in 20 (87%) of 23 patients in the ultrasound and 12 (50%) of 24 patients in the landmark group (RR = 1.74; 95% CI = 1.13 to 2.67; *P* = 0.006). Appropriate longitudinal placement was achieved in 21 (95%) of 22 in the ultrasound and in 21 (95%) of 22 in the landmark group (RR = 1.00; 95% CI = 0.88 to 1.14; *P* = 1.00). Longitudinal position could not be determined, owing to severe tracheitis in two patients in the landmark group and one patient in the ultrasound group. First-pass success rate was 20 (87%) of 23 in the ultrasound group and 14 (58%) of 24 in the landmark group (RR = 1.49; 95% CI = 1.03 to 2.17; *P* = 0.028). Reduction in complication rate was not statistically significant: 5 (22%) of 23 in the ultrasound group compared to 9 (37%) of 24 in the landmark group (RR = 0.58; 95% CI = 0.23 to 1.47; *P* = 0.24). Discounting ‘minor bleeding not requiring intervention’, the complication rates were 2 (9%) of 23 in the ultrasound group and 2 (8%) of 24 in the landmark group, which are comparable to those reported in the literature. One accidental decannulation and one pressure ulcer were noted in the ultrasound group during the follow-up period. There were no serious intermediate-term complications (Table 2). There were four deaths in each group, all related to the underlying disease process.

## Discussion

Ultrasound guidance significantly improved the rate of first-pass puncture and puncture accuracy.

**Figure 3:**
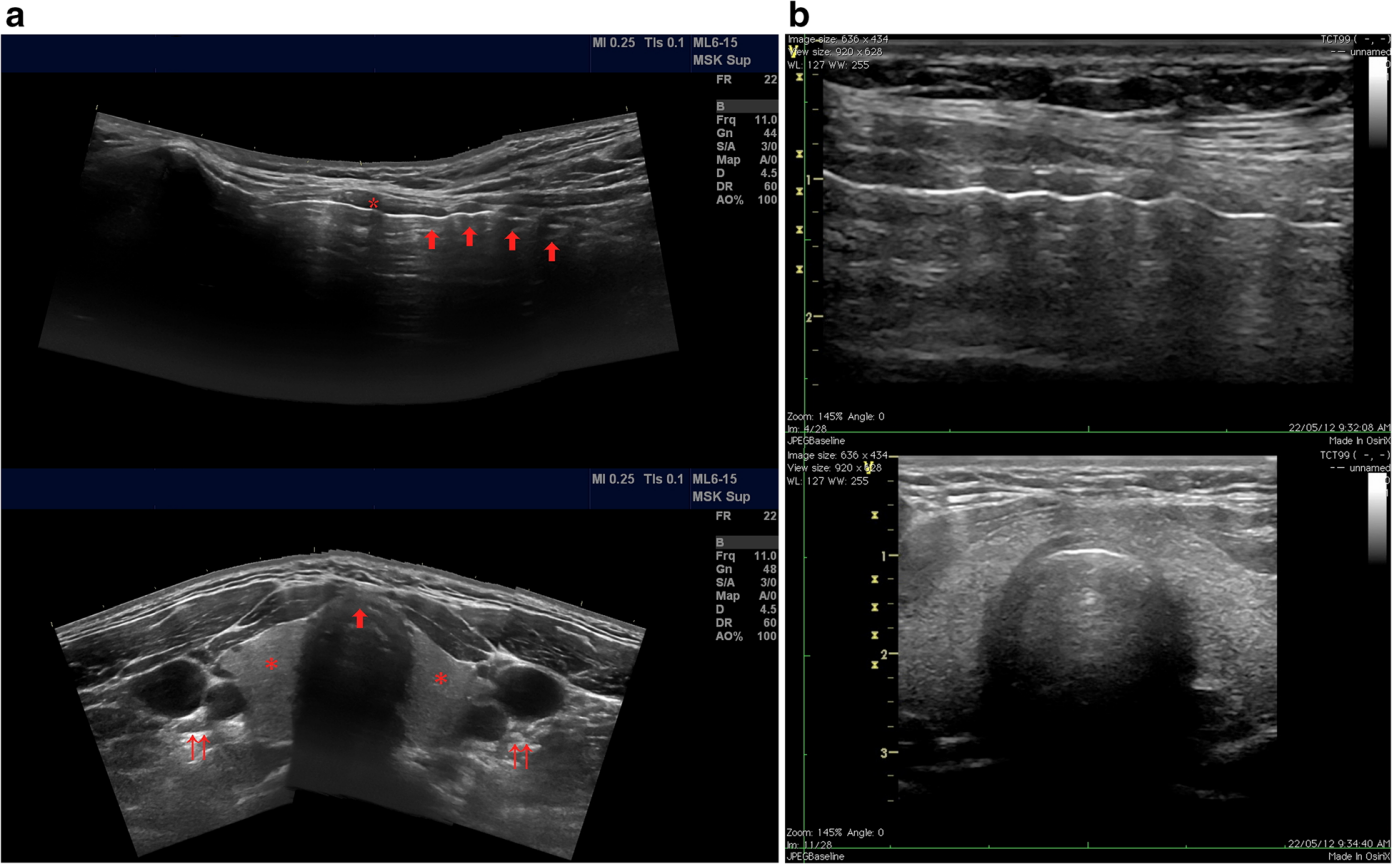
**Ultrasound anatomy of the neck. (a)** *Top*: Panoramic longitudinal view. The asterisk indicates the cricoid cartilage, and the arrows point to the tracheal rings. *Bottom*: Panoramic transverse view. The asterisk indicates the thyroid gland, the double-arrows point to the left and right carotid arteries and the jugular vein, and the single arrow points to the tracheal cartilage, ideal puncture site in the anterior midline. **(b)** Actual longitudinal (*top*) and transverse (*bottom*) views obtained with a linear probe during bedside percutaneous tracheostomy. Panoramic ultrasound images courtesy of James Rippey.

The ultrasonographic anatomy of the anterior neck prior to tracheostomy was first described in 1995 [14], and a report of real-time ultrasound-guided puncture for PDT was first published in 1999 [15]. Two-dimensional ultrasound using a linear array probe readily identifies the position and anatomical relation of important landmarks. These include the thyroid and cricoid cartilage, the tracheal rings, the thyroid gland and the carotid and jugular vessels (Figures 3a and 3b). Aberrant vascular structures crossing the midline can further be evaluated by colour or spectral Doppler. Real-time imaging can be used to identify the desired level of puncture in a sagittal plane in the midline over the trachea, and a 90° rotation of the probe allows for an out-of-plane approach to guiding the needle (represented by an acoustic shadow) towards the midline. Care should be taken because the needle must enter the trachea almost directly below the puncture site, making the angle of insonation of the needle low, resulting in potential difficulties in identifying and discerning the needle tip from the shaft. Indirect ultrasonographic signs of the needle advancement, such as indentation of the tissues, can be used as additional guidance to direct the needle towards the anterior tracheal midline (see movie file in Additional file 1).

The theoretical advantage of using preprocedural ultrasound lies with the ability to identify aberrant pretracheal vasculature to avoid immediate vascular complications [6]. It may also aid in proper selection of tracheostomy tube size and length, especially in patients with an increased pretracheal soft-tissue diameter or in children [16]. Intraprocedural ultrasound may assist not only with identifying the tracheal anatomy and potentially aberrant vessels but also with identifying the preferred puncture location and guiding the needle puncture of the trachea in real time, not dissimilar to the technique routinely used in ultrasound-guided vascular access.

It is common for patients receiving a tracheostomy to have anatomy that is considered ‘difficult’. This is most often due to obesity or to deformity from chronic musculoskeletal pathology or prior injuries and surgical procedures. The difficulties encountered in locating landmarks such as the cricothyroid membrane and difficulties with tracheal puncture when the tracheal anatomy is not readily palpable have been highlighted in multiple recent studies, both in real patients and on simulated models [17,18]. Ultrasound has been successfully used in simulated models and difficult clinical cases to guide the tracheal puncture [19,20].

The finding that real-time ultrasound-guided tracheal puncture has a significantly higher first-pass success rate has important implications, not only in elective surgery but also in emergency airway procedures. A number of currently used percutaneous emergency airway devices rely on tracheal puncture, and as these are reserved for ‘can’t intubate, can’t ventilate’ scenarios, rapid and reliable access to the airway in these circumstances is of paramount importance.

To date, there have been no studies in which investigators have reported the incidence and degree of inappropriately positioned tracheal punctures. To our knowledge, we are the first group to highlight this common occurrence during tracheal puncture guided by landmark anatomy. The extent to which deviation from the midline will influence the development of complications, either in the periprocedural setting or later on, is also unknown. Vascular complications are relatively easy to detect, but it is difficult to quantify their severity. Demonstration of the true incidence of long-term complications such as tracheal stenosis is difficult and often considered impossible in a study cohort, owing to the nature of the investigations required (for example, computed tomography or endoscopy). The rarity of serious or fatal complications makes a study powered to detect a significant difference in this regard impractical. However, there are plausible theoretical grounds and a large amount of observational data in the literature suggesting that multiple complications can potentially be avoided by an appropriately positioned tracheal puncture.

**Figure 4:**
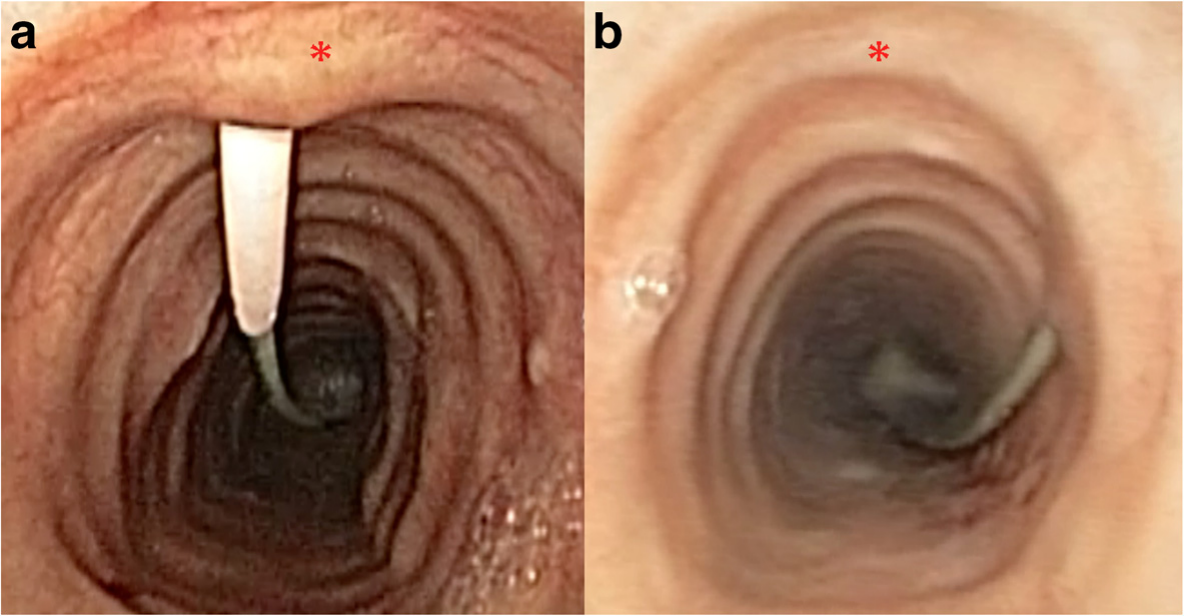
**Bronchoscopic views.** Appropriately positioned tracheal puncture **(a)** and an example of extremely lateral puncture with potential for complications **(b)**. The asterisks mark the anterior tracheal midline.

**Figure 5:**
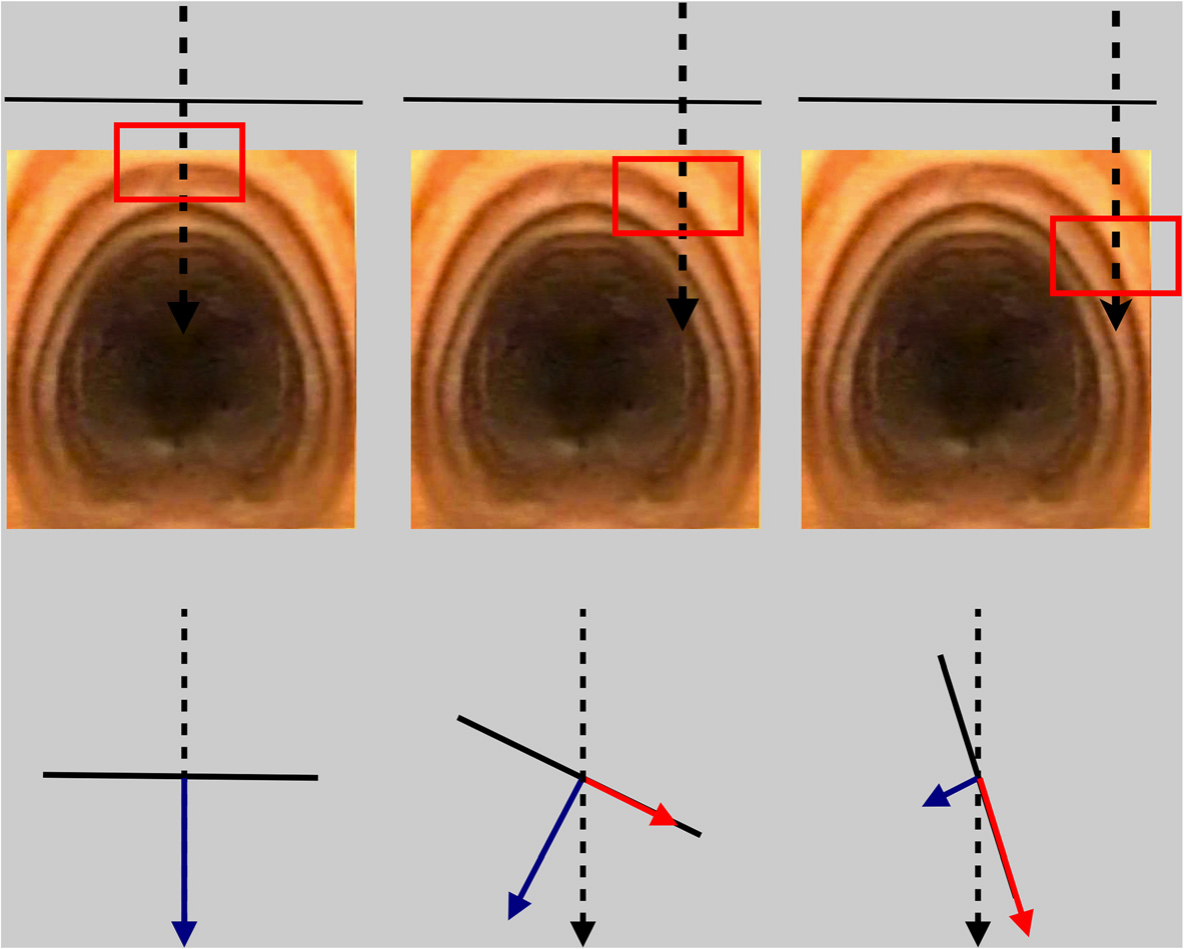
**Risk associated with lateral tracheal puncture.** Downward force (dashed black arrow) applied during dilation of the tract can be broken up into force vectors which are perpendicular (blue arrow) and parallel (red arrow) to the tracheal wall (black line). At a 90° angle, all of the force is perpendicular. With the angle becoming more oblique, hitting the lateral curve of the tracheal wall, there is an increasing proportion of the downward force which is parallel to the wall and a decreasing proportion directed towards the tracheal lumen. This can lead to bending of the guidewire and subsequent paratracheal tract dilation or tearing of the tracheal wall itself.

There can be little doubt that a significant proportion of serious complications in the immediate periprocedural setting relates to unanticipated anatomical variation in the vasculature [4]. Ultrasound has a sound theoretical benefit in this setting, and a number of authors have reported changing the planned puncture location based on ultrasound findings in up to as many as 50% of their patients [21-23]. During dilation of the tract, laterally positioned punctures also change the force vectors resulting from applying downward pressure onto an oblique section of the anterolateral trachea. A significant amount of force can thus be directed parallel rather than perpendicular to the tracheal wall (Figures 4 and 5). This can be particularly dangerous, as it predisposes to bending of the guidewire and subsequent paratracheal dilatation of the tract, or tearing of the tracheal wall by the dilator itself. An appropriately positioned midline puncture can potentially prevent these complications.

The development of late complications can also be influenced by initial tracheostomy tube position. Published cases of serious late bleeding complications have often arisen in the setting of very laterally or caudally placed tracheostomy tubes, which eventually eroded into vascular structures [5]. Other long-term complications, such as tracheal stenosis and intermittent tracheostomy tube obstruction contributing to failed ventilator wean, have also been reported as consequences of inappropriate tracheostomy tube position [24-26]. Appropriate midline and longitudinal puncture position can therefore also have implications in reducing late complication rates.

In summary, although this study was not powered to detect a difference in the rate of complications, there is sufficient evidence in the literature to support a link between appropriate tracheal puncture position and decreased complication rates, both in the immediate procedural setting and in the long term.

We conducted a prospective randomised controlled trial to study the utilisation of real-time ultrasound to guide tracheal puncture during PDT in a representative cohort of general adult intensive care patients. Ultrasound is generally available in ICUs, and many consider real-time ultrasound mandatory for certain procedures, including central venous cannulation and thoracocentesis. It was therefore reasonable to study the use of real-time ultrasound to guide tracheal puncture during PDT, as this technique can be broadly generalised.

### Strengths and limitations

The strengths of the study include prospective randomisation, strict maintenance of allocation concealment, blinded assessment of primary outcome data, limited exclusion criteria and a high percentage of capture of eligible patients during the study period. Pragmatic inclusion of a representative cohort of proceduralists with various levels of expertise and training makes the data generalizable beyond the involved institutions. The endpoints were clearly defined, simple and clinically relevant, and data were analysed using straightforward statistical methods.

Potential limitations of the study include an operator skill mix biased towards the landmark technique, in which most operators had significantly more experience compared to the ultrasound technique. This may have led to an underestimation of the effect size. We examined the use of a strict percutaneous tracheal puncture technique which may not be the method of choice in all institutions. The possible additional utility of ultrasound guidance is unclear if deep dissection is performed before the tracheal puncture or if bronchoscopy is used during the needle puncture to guide the needle. The number of puncture attempts was self-reported, and some procedural complications, such as ‘minor bleeding not requiring intervention’, are difficult to define and can be subjective. Serious adverse events are rare, and potential differences in length of ventilation or intensive care stay are likely to be small; therefore, this study was not powered to demonstrate a difference in these outcome measures. Craniocaudal placement of the tracheal puncture was difficult or impossible to determine in a small number of cases, such as in patients with significant tracheitis. There was a small apparent difference in baseline body mass index between the two groups, and although it has been demonstrated that morbid obesity does not influence the safety or efficacy of PDT when ultrasound is used [23], there are no reports in the literature to date describing whether a difference exists when the landmark technique is utilised. The potentially incremental benefit of ultrasound over the landmark technique in the morbidly obese remains to be investigated.

## Conclusions

Ultrasound-guided tracheal puncture, in comparison to the landmark technique, is more efficient and is associated with a significantly higher first-pass success rate. Ultrasound-guided puncture is also more accurate than the landmark technique and results in a significantly higher rate of appropriate midline punctures. Fewer procedural complications were observed in the ultrasound group; however, this difference did not reach statistical significance. The present data support wider routine use of real-time ultrasound guidance for tracheal puncture during percutaneous tracheostomy.

## Key messages

- Real-time ultrasound guidance improves the accuracy of tracheal needle puncture during PDT, thereby potentially decreasing both short- and long-term complication rates.
- Real-time ultrasound guidance improves the first-pass success rate of tracheal needle puncture during PDT, highlighting its potential utility during time-critical airway procedures.
- Fewer procedural complications were observed with the use of ultrasound guidance; however, this study was not powered to detect a statistically significant difference.

### Consent

Written informed consent was obtained from the patients or their relatives for publication of this manuscript and accompanying images. A copy of the written consent is available for review by the Editor-in-Chief of this journal.

## Additional file

Additional file 1:
**Ultrasound anatomy of the neck and real-time, ultrasound-guided tracheal puncture.** The panoramic ultrasound images demonstrate the ultrasound anatomy of the neck in relation to performing a percutaneous dilatational tracheostomy. In the transverse view one can identify the tracheal cartilage (yellow), thyroid gland (orange) and carotid and jugular vessels (red and blue respectively). Rotating the probe 90 degrees yields a longitudinal view where the thyroid, cricoid and tracheal ring cartilages (yellow) are identified in cross section. During the procedure a longitudinal view allows identification of the cricoid and tracheal rings (yellow) and the desired puncture level between the 1st and 4th tracheal rings. Rotation to 90 degrees and scanning cranially and caudally allows for visualisation of potential aberrant midline vascular structures as well as identification of important landmarks such as the thyroid cartilage and vocal cords (yellow, blue), the cricoid cartilage (yellow) and the tracheal rings with the thyroid gland and isthmus lying anteriorly (yellow, orange). The needle path is visualised in real time represented by an acoustic shadow. Indentation of the tracheal ring by the needle tip can also be observed. Penetration of the tracheal lumen can be confirmed by demonstrating air escaping through the cannula. The guide wire is inserted and after bronchoscopic confirmation of intraluminal placement dilation of the tract and insertion of the tracheostomy tube follows as per standard procedure. Footnote: The patient depicted in this video is not a study participant. Consent for recording and educational use, including publication, was obtained. Panoramic images courtesy of Associate Professor James Rippey. 3D human anatomy graphics created and used with permission Visible Body 3D Human Anatomy Atlas. (www.visiblebody.com).

## Abbreviations

PDT: Percutaneous dilatational tracheostomy
